# Clinical Management of Patients with First-Episode Atrial Fibrillation Detected in the Acute Phase of Myocardial Infarction

**DOI:** 10.36660/abc.20190733

**Published:** 2019-11

**Authors:** Mauricio Scanavacca, Tan Chen Wu

**Affiliations:** 1 Universidade de São Paulo - Faculdade de Medicina Hospital das Clínicas - Instituto do Coração - Cardiologia, São Paulo SP - Brazil

**Keywords:** Atrial Fibrillation/complications, Myocardial Infarction/complications, Ventricular Dysfunction, Anthypertensive Agents, Anticoagulants, Stroke

Atrial fibrillation (AF) episodes have been traditionally observed in up to 20% of the patients who suffer an acute myocardial infarction (AMI); 5-10% of them, as a “first episode”, during hospital admission.^1^ The etiology of AF, in this phase of AMI, includes several factors, such as elevated atrial pressure resulting from acute ventricular dysfunction, associated atrial ischemia (more common in inferior wall AMI), secondary inflammatory reaction, changes in autonomic nervous system behavior and in the neurohumoral pattern related to the pathophysiology of AMI, especially in those with ventricular dysfunction.^1-3^

A recent study published in the Arquivos Brasileiros de Cardiologia by Congo et al.^4^ suggest that, in the last years, the incidence of AF in the acute phase of AMI may be decreasing, due to the greater access of patients to early reperfusion and to better clinical treatment, associated with the more frequent use of angiotensin converting enzyme inhibitors (ACE inhibitors), beta blockers, statins and antiplatelet therapy. In addition, the study corroborates previous observations that new onset AF is associated to poorer clinical evolution and raises awareness of the clinical management of these patients, in particular in relation to the non-use of oral anticoagulants at hospital discharge.

The study was performed using data obtained from a Portuguese national registry on AMI, which included 6,325 patients from 2010 to 2017, out of whom 365 (5.8%) presented an initial episode or first incident AF in the acute phase of AMI, most of them in the first day of hospitalization. Patients admitted due to AMI and ST-segment elevation who had (first episode) AF were compared with patients who did not develop AF during hospitalization.

Acute reperfusion rates and strategies were similar in both groups (without AF = 83.2%, with AF = 82.9%), apparently with no impact on the occurrence of AF. However, it was observed that patients with AF had longer door-to-balloon time, less prescription of beta blockers, ACE inhibitors and angiotensin receptor blockers. Factors like advanced age, previous stroke, complete atrioventricular blockage and inferior infarction were independent predictors for the occurrence of AF.

The occurrence of AF was associated with longer hospitalization time, higher incidence of complications and mortality (13.4%) compared with the control group (3.8%; p < 0.001). However, the occurrence of AF was not an independent predictor of in-hospital mortality, which suggests that it may be only an indicator of greater disease severity.

Unfortunately, the study does not provide information on the clinical presentation of AF, detailed clinical management of patients during hospitalization and AF recurrence during evolution. Two-thirds of the patients who developed new onset AF received amiodarone during hospitalization, which was maintained in 26% of patients at hospital discharge. In addition, only 20.6% were discharged on oral anticoagulants, with or without double platelet aggregation. These observation may reflect the lack of information on the literature in how to manage these patients. Then, more robust data on the decisions concerning heart rate (HR) control or rhythm control and the need for maintenance of anticoagulation after hospital discharge are still required.

For instance, in the VALsartan In Acute myocardial iNfarcTion (VALIANT) trial,^5^ 1,131 patients who presented with AF after AMI and heart failure were assessed; 371 were treated with rhythm control (amiodarone 87.3%) and 760 patients with HF control strategy. After adjustment to the clinical profile of patients, rhythm control was associated with increased mortality in the first 45 days (HR: 1.9, 95% CI 1.2-3.0, p = 0.004), but not with late mortality. In this study, mortality cannot be attributed to the use of Class I antiarrhythmics (AA), since most of deaths occurred in the *rhythm control group* receiving amiodarone (95.6%). The relation between amiodarone and mortality was also observed in the Global Use of Strategies to Open Occluded Coronary Arteries (GUSTO-3) trial.^6^ For rhythm control, 132 patients (12%) used Class I AA agents, 55 (5%) sotalol and 168 (15%) amiodarone. The sinus rhythm was restored in 72% of the patients who received Class I drugs, in 67% of those receiving sotalol, and in 79% of the patients receiving amiodarone. There was a tendency to less mortality with the use of Class I AA drugs and sotalol in relation to the patients receiving amiodarone, which the authors attributed to the probable option for Class I AA drugs and sotalol in the case of patients with better clinical condition.

The Global Utilization of Streptokinase and TPA for Occluded Coronary Arteries *(*GUSTO-I) trial,^7^ on the other hand, which included 40,891 patients with AMI, registered an increased incidence of stroke in patients with AF in the acute phase of AMI (3.1%), compared with those who maintained the sinus rhythm (1.3%) - (p = 0.0001). Ischemic strokes were observed in 1.8% and 0.5% of patients with or without AF, respectively. In addition, the this study did not provide data on the use of anticoagulants after hospital discharge. Hence, Siu et al.^8^ analyzed 431 consecutive patients with inferior wall AMI and preserved ventricular function. They found that transient new-onset AF during hospitalization was observed in 59 patients (13.9%). Over a mean follow-up period of one year, the incidence of AF was of 22% in patients with transitory AF in admission, compared with 1.3% (p = 0.01) in the group without AF and, even more importantly, the incidence of ischemic stroke was of 10.2% in patients with transitory AF and of 1.8% in those without AF (p = 0.01). It is worth highlighting that the patients with AF did not receive anticoagulants, only antiplatelet agents. Similar findings were obtained in the Optimal Trial in Myocardial Infarction with the Angiotensin II Antagonist Losartan (OPTIMAAL) trial^9^ in patients with AMI and ventricular dysfunction, in which the presence of new onset AF was also associated with increased risk of stroke in the first 30 days (HR 14.6, p < 0.001) and during the whole period of observation of the study (HR 2.29, p < 0.001).

Current guidelines recommend that patients with acute coronary syndrome and AF should receive anticoagulant therapy (warfarin or NOACs), associated with aspirin and/or clopidogrel for at least 6 months, except if they are absolutely contraindicated.^10,11^ However, the studies that support it are based on the existence of known AF or AF prior to the coronary event.^12-17^

In the absence of randomized clinical trials, it seems reasonable that this recommendation should also be applied to the patients who presented with a first-episode AF in the acute phase of AMI, particularly in those with high-risk predictors of recurrence and with elevated CHA_2_DS_2-_VASc score^18^.
